# Amino Acids and TOR Signaling Promote Prothoracic Gland Growth and the Initiation of Larval Molts in the Tobacco Hornworm *Manduca sexta*

**DOI:** 10.1371/journal.pone.0044429

**Published:** 2012-09-12

**Authors:** Karen Kemirembe, Kate Liebmann, Abigail Bootes, Wendy A. Smith, Yuichiro Suzuki

**Affiliations:** 1 Department of Biological Sciences, Wellesley College, Wellesley, Massachusetts, United States of America; 2 Department of Biology, Northeastern University, Boston, Massachusetts, United States of America; Michigan State University, United States of America

## Abstract

Molting in arthropods is orchestrated by a series of endocrine changes that occur towards the end of an instar. However, little is understood about the mechanisms that trigger these endocrine changes. Here, nutritional inputs were manipulated to investigate the minimal nutritional inputs required for a *Manduca sexta* larva to initiate a molt. Amino acids were found to be necessary for a larva to molt, indicating the involvement of an amino acid sensitive pathway. Feeding rapamycin, an inhibitor of the target of rapamycin (TOR) signaling, delayed the onset of a molt and resulted in abnormally larger larvae. Rapamycin also suppressed the growth of the prothoracic glands relative to the whole body growth, and this was accompanied by suppression of ecdysone production and secretion. Higher doses of rapamycin also slowed the growth rate, indicating that TOR signaling also plays a role in systemic growth. TOR signaling therefore couples the nutritional status of the larva to the endocrine system to regulate the timing of a molt.

## Introduction

The proximate neuroendocrine mechanisms underlying major developmental transitions are well studied in many species [1]. However, these mechanisms are often set in motion once the organism attains a certain physiological state [2], and how the physiological state is relayed to neuroendocrine centers remains poorly understood. For instance, we know which neuroendocrine regulators are involved during molting and metamorphosis in insects and puberty in humans [3], [4], [5], [6]. Yet, how the neuroendocrine centers integrate internal and external cues to determine when to initiate major developmental changes is only beginning to be understood.

Ecdysozoans have a hard exoskeleton that must be shed to allow for growth. This shedding of the cuticle is called molting. The molts occur at regular intervals throughout the juvenile phase and also during the adult phase in some species [7]. Each molt is accompanied by dramatic hormonal changes that initiate production of molting fluid, synthesis of a new cuticle and eventual ecdysis [8].

Much is known about the endocrine changes associated with a molt. The primary hormones that coordinate molting events in insects are the arthropod steroid hormones, ecdysteroids. Several different types of ecdysteroids exist, but among lepidopterans, the primary edysteroid responsible for molting is 20-hydroxyecdysone (20E) [9]. 2-dehydroecdysone and 3-dehydroecdysone are secreted from the prothoracic glands in response to prothoracicotropic hormone (PTTH), a neuropeptide synthesized in the brain and released from the corpus cardiacum/corpora allata [6], [9], [10], 11,12. In the hemolymph, 2-dehydroecdysone and 3-dehydroecdysone are rapidly converted to ecdysone, which is subsequently converted to 20E, a more active form of ecdysone that initiates a series of events associated with molting and eventual ecdysis of the larva [8], [9], [13], [14].

Relatively little is known, however, about the mechanism by which the dramatic hormonal changes are initiated in the first place. In hemipterans, such as *Oncopeltus* and *Rhodnius*, stretch receptors in the abdomen cue the CNS to initiate a molt [15], [16]. However, in other insects, the trigger for molting does not appear to be linked to stretch receptors. A recent study has shown that the timing of a molt can be influenced by oxygen concentrations in the atmosphere and that oxygen limitation might be a major cue that triggers endocrine changes associated with a molt [17]. However, the mechanism by which the oxygen level is relayed to the neuroendocrine centers remains unknown.

Recent studies suggest that in addition to oxygen levels, nutrients might also play key roles in influencing the timing of ecdysone-mediated developmental events. The critical weight is defined as a weight at which the larva can pupate without delay even under starvation conditions [18], [19]. Studies in *Drosophila* have shown that the prothoracic glands grow during the final instar and that the critical weight corresponds to the size at which the prothoracic gland attains a particular threshold size [20]. This prothoracic gland growth in *Drosophila* is nutrient dependent. When the prothoracic gland size is artificially increased by modulating insulin signaling, a nutrition-sensitive pathway, the critical weight decreases such that the animals pupariate at a smaller body size, whereas decreasing the size of the prothoracic glands causes the animals to pupariate at larger sizes [20], [21], [22]. However, insulin signaling alone is insufficient to increase ecdysteroidogenesis/ecdysteroid secretion in early fifth *Manduca sexta* larvae [23]. Several recent studies indicate that nutrition also has direct effects on ecdysteroidogenesis in the prothoracic glands during the final instar. In particular, target of rapamycin (TOR) signaling, an amino acid-responsive signaling pathway, appears to play a critical role in ecdysteroidogenesis [24], [25]. Thus, nutrition-dependent prothoracic gland growth and activity appears to be critical in determining the timing of the initiation of metamorphosis.

Here, we investigated the role of nutrients on the growth of prothoracic glands and molting in *M. sexta*. Amino acids were found to be essential for molting. We focused on the role of TOR signaling pathway and found that it plays a major role in regulating the timing of a molt. Feeding rapamycin, an inhibitor of TOR signaling, also led to reduced ecdysteroidogenesis and ecdysone titers.

## Methods

### Animal Husbandry

*M. sexta* colony was established from the University of Washington colony. Larvae were maintained at 26°C and 18:6 hr light:dark cycle. Larvae were maintained in individual cups on artificial diet until the time of experiment. The timing of the molt was investigated in the penultimate instar (fourth instar) because an additional endocrine regulator, juvenile hormone, determines the critical weight in the fifth instar [26]. The molting in earlier instars likely relies only on secretion of ecdysone.

### Diet Treatments

Larvae weighing 0.17–0.21g were removed from normal artificial diet at the end of the third instar once the larvae started to exhibit signs of head capsule slippage. The larvae were placed on non-nutritive diet consisting of agar, vitamins and minerals only to ensure that all larvae started to feed on the experimental diet at the same time. Two days later, larvae were transferred to the experimental diets. In all experimental diets, wheat germ, yeast, cholesterol and linseed oil were not added to remove the confounding effects of lipid consumption. To initially determine the roles of amino acids and sugars on the timing of a molt, larvae were fed on 2%, 4%, 5.8% or 8% casein and 3%, 5%, 7% or 9% sucrose diets (Table S1). To ensure that any delays in the timing of larval molt were not due to an energy deficiency, the decreased casein was replaced with an amount of sugar with the equivalent energy. Both casein and sucrose were estimated to provide 4 kcal/g of energy. For diluted diet experiments, 40%, 60% or 100% normal diets were used as described previously [27]; Table S1). Diets were replenished as needed.

### 20-hydroxyecdysone (20E) Injections

To determine if nutrients modulate the secretion of or sensitivity to ecdysteroids, 20E was injected into starved larvae. Larvae weighing 0.17–0.21g were starved for two days at the onset of head capsule slippage as described above. Larvae were then injected with 5 µl of 5 mg/ml 20-hydroxyecdysone (Sigma) dissolved in water every 12 hrs via the prolegs. The timing of apolysis was recorded. Control larvae were injected with the same amount of water instead of 20E.

### Rapamycin Treatments

To investigate the effects of rapamycin on molting and prothoracic gland growth, larvae weighing 0.17–0.21g were starved for two days on starvation diet as described above and then fed a diet treated with rapamycin (LC Labs). Rapamycin was dissolved in DMSO at various concentrations and 20 µl of this solution was added to 180 µl of phosphate buffered saline (PBS; 0.15 M NaCl, 0.0038 M NaH_2_PO_4_, 0.0162 M Na_2_HPO_4_; pH 7.4). The mixture was mixed completely, homogenized and pipetted evenly onto 1g of thinly sliced normal artificial diet. One gram of similarly treated diet was added every day. Controls consisted of 20 µl of DMSO dissolved in 180 µl of PBS instead of the rapamycin mixture. Signs of head capsule slippage or spiracular apolysis were used as markers for the onset of a molt.

### Prothoracic Gland Dissections and Measurements

Prothoracic glands were dissected out in PBS from anesthetized larvae immediately after weighing. The glands were fixed in 3.7% formaldehyde overnight and then mounted in 80% glycerol. The glands were imaged with an 18.2 Color Mosaic camera (Diagnostic Instruments) mounted on a Nikon 50i Trinocular Microscope and SPOT Advanced software. For each prothoracic gland, the cross-sectional areas of 10 cells were measured in pixels and averaged using ImageJ (NIH Image).

### Ecdysone Measurements

Hemolymph ecdysteroid levels, and ecdysone secretion by isolated prothoracic glands, were determined by radioimmunoassay as previously described [23], [28]. [^3^H]Ecdysone was obtained from PerkinElmer, and anti-ecdysone antibody was a generous gift from Dr. Lawrence Gilbert. The antibody cross-reacts with both ecdysone and 20-hydroxyecdysone as previously described [28]. Hemolymph samples were collected by removal of a proleg from anesthetized larvae. Hemolymph samples were extracted with an equal volume of methanol, and the equivalent of 2.5 ml hemolymph was subjected to radioimmunoassay. *In vitro* ecdysone secretion was determined by incubating pairs of prothoracic glands in 30 ml Grace’s culture medium at room temperature for 2 hr and assaying medium for secreted ecdysone.

### Western Blots

To investigate whether nutrition influences targets of TOR signaling, levels of phosphorylated 4E-binding protein (4E-BP, a downstream target of TOR signaling) were compared for animals fed a non-nutritive, 8% sucrose only or 8% casein only diet. Prothoracic glands were dissected in PBS, placed into 2X sample buffer and subsequently boiled for 5 min. Four pairs from casein-fed larvae and eight/nine pairs from starved and sucrose-fed larvae were pooled per sample. The amount of total protein was determined using bicinchoninic acid assay (Pierce) and equal amounts were loaded for gel electrophoresis. Fat body from a single larva was dissected and flash frozen. Total protein levels were determined, and equal amounts were boiled in 2X sample buffer and subsequently loaded for gel electrophoresis. The blots were probed using anti beta-tubulin (Developmental Studies Hybridoma Bank), anti phospho-4E-BP (Cell Signaling Technologies) and anti non-phospho-4E-BP (Cell Signaling Technologies) antibodies. The blots were visualized with ECL Plus or ECL Western Blotting Detection Reagent (GE Healthcare Life Sciences) following incubation with horseradish peroxidase-conjugated secondary antibodies.

## Results

### Amino Acids are Required for Molting in Manduca Sexta

**Figure 1:**
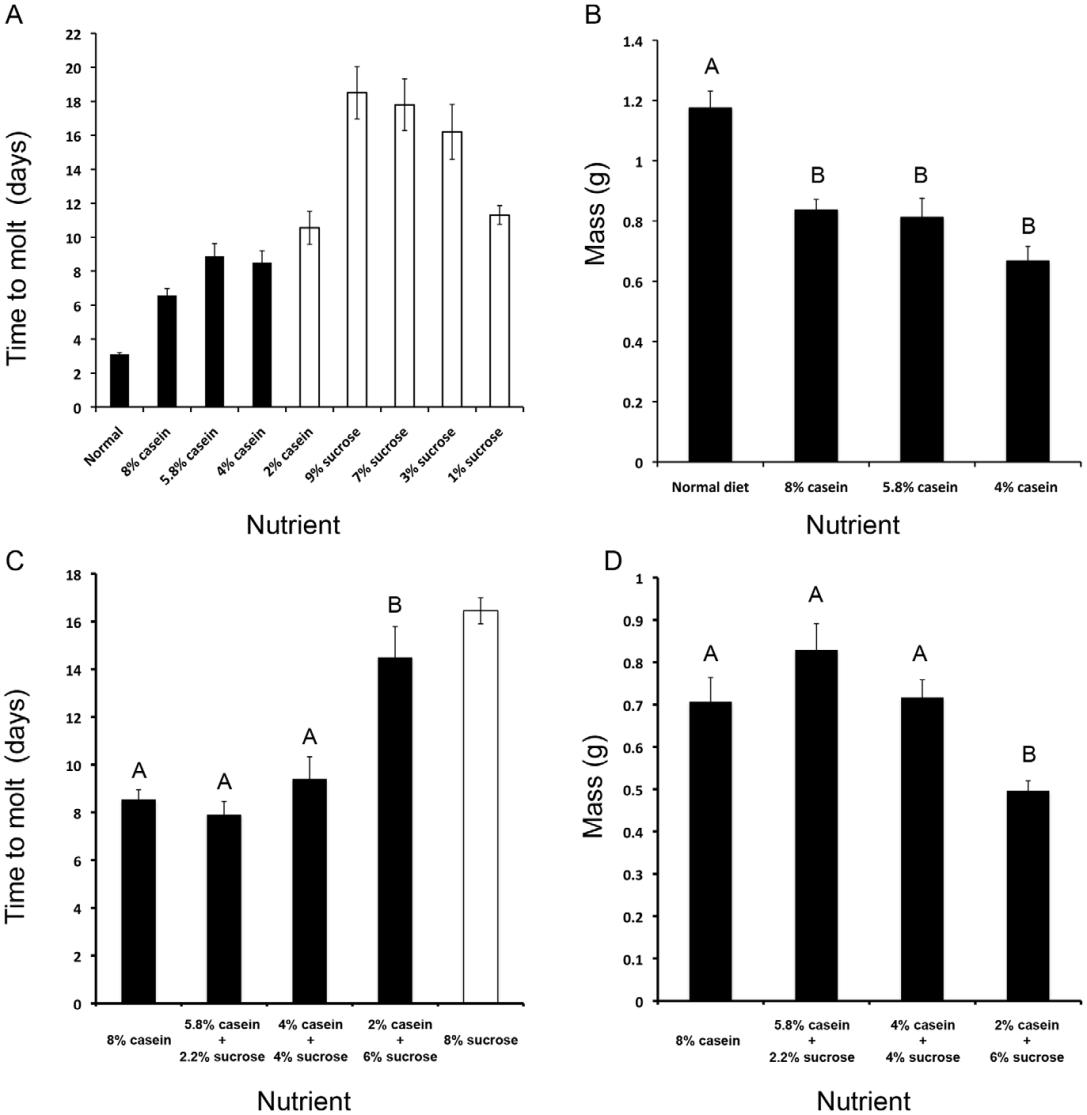
Effect of various nutrients on the survival and time to molt. (A) Time to molt (black bars) or death (open bars) in 4^th^ instar larvae fed various concentrations of casein or sucrose only diets (n = 8–12 for each treatment). (B) Final size at the time of the molt for larvae fed normal or casein-only diets. Different letters represent statistically significant differences (ANOVA with Tukey-Kramer HSD test; p<0.0001). The normal diet fed larvae were significantly larger than those fed 4% (p<0.0001), 5.8% (p = 0.0001) or 8% (p = 0.0001) casein only diets. The various casein diets did not produce significantly different effects on the final size at the time of molt (n = 8–12 for each treatment). Error bars represent standard errors. (C) Time to molt (black bars) or death (open bars) in 4^th^ instar larvae fed casein diets supplemented with sucrose (n = 10–11 for each treatment). Different letters represent statistically significant differences (ANOVA with Tukey-Kramer HSD test; p<0.0001). Molting time of larvae fed a 2% casein diet supplemented with 6% sucrose was significantly delayed relative to the 8% casein only, 5.8% casein/2.2% sucrose and 4% casein/4% sucrose diets (p<0.0001, p<0.0001 and p = 0.001, respectively). (D) Final size at the time of the molt for larvae fed casein diets supplemented with sucrose. Different letters represent statistically significant differences (ANOVA with Tukey-Kramer HSD test; p = 0.0007). Larvae fed a 2% casein diet supplemented with 6% sucrose molted at a significantly smaller size relative to the 8% casein only, 5.8% casein/2.2% sucrose and 4% casein/4% sucrose diets (p = 0.0352, p = 0.0003 and p = 0.0247, respectively).

In order to determine the minimum nutritional inputs required for the fourth instar *M. sexta* to molt to a fifth, larvae were fed diets containing varying concentrations of either sucrose or casein. A diet containing 4% or more casein alone was sufficient to induce a molt (Fig. 1A). Larvae fed casein alone molted at a significantly lower weight relative to the normal diet-fed larvae (Fig. 1B). Larvae fed on sucrose alone did not molt even though they survived on average for 18 days. These results indicate the amino acids were required for the larva to initiate a molt.

To ascertain that the delay or lack of a molt was in fact due to a limitation in amino acids rather than a shortage in energy supply with decreased amino acids, each decrease in amino acids was replaced by an energetically similar amount of sucrose. It was observed that reduced amounts of casein still led to a delay in the timing of a molt (Fig. 1C). Larvae fed on 2% casein supplemented with sucrose were eventually able to initiate a molt despite a delay, unlike those fed 2% casein alone (Fig. 1A, C). Presumably, the presence of sucrose provides enough energy to keep the larvae alive until they can initiate a molt. In larvae fed 2% casein supplemented with sucrose, the larvae molted at a smaller size compared to other larvae (p<0.05; Fig. 1D). Taken together, when the amount of amino acid was limited, the timing of a molt was delayed even when the total energetic value of the diet was controlled for, indicating that amino acids are necessary for initiating a molt.

### Initial Feeding of Sucrose before Amino Acids Shortens the Timing of the Molt

**Figure 2:**
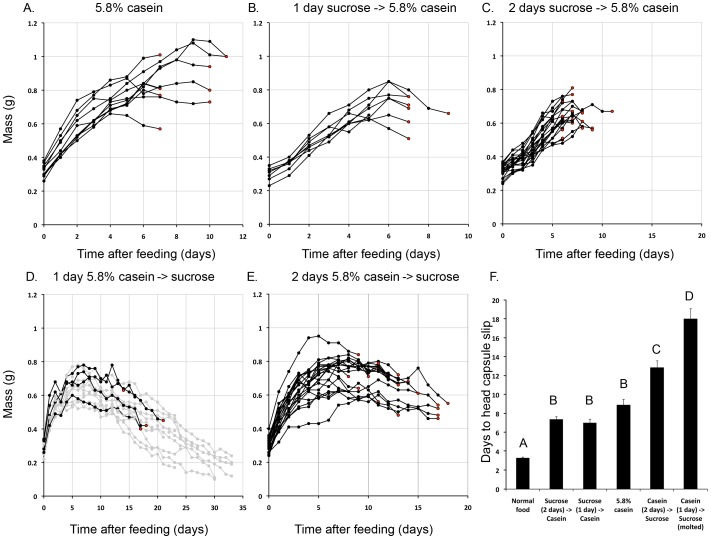
Growth of fourth instar *M. sexta* larvae transferred from sucrose to casein diets and vice versa. (A–E) Growth trajectory of fourth instar larvae transferred from sucrose to casein diets and vice versa. Day 0 larvae were fed 5.8% casein and 7% sucrose in the specified order, and their growth trajectory was monitored. The red dots indicate the timing of head capsule slippage (n = 8–20 for each treatment). Black circles indicate the animals that molted whereas grey circles represent the animals that ultimately died without molting. (F) The time taken for larvae to initiate a molt. Different letters represent statistically significant differences (ANOVA with Tukey-Kramer HSD test; p<0.05; n = 4–20). p<0.0001 for all statistically different pairwise comparisons except between larvae fed on casein for either one or two days and transferred to sucrose (p = 0.0002), and between those fed on normal diet and those fed on sucrose for one day followed by casein (p = 0.0008). Error bars represent standard errors.

To investigate the importance of sucrose as a complement to amino acids in regulating the timing of the molt, fourth instar larvae were fed sucrose-only diets before or after transfer to a casein-only diet and monitored for the time to molt. Most of the larvae fed on casein alone molted on average after nine days (Fig. 2A, F), whereas those fed on sucrose for one or two days and then transferred to a casein diet molted after only seven days at significantly smaller body sizes (Fig. 2B, C, F; p = 0.0051 and p = 0.0001, respectively; ANOVA with Tukey-Kramer HSD test). Interestingly, when the order of feeding was reversed and larvae were fed casein for one or two days before switching to a sucrose only diet, all larvae that were fed casein for two days were able to initiate a molt (Fig. 2E). A few of the larvae fed on casein for one day initiated a molt but at a much later time (Fig. 2D, F). Molting in these 1-day casein fed larvae was not observed until about 18 days later. In both of these treatments, larvae molted at a significantly smaller sizes compared to those that were fed casein only diets continuously (p<0.0001 and p = 0.0002 for larvae fed casein for one and two days before transfer, respectively; ANOVA with Tukey-Kramer HSD test). These findings suggest that one day of amino acid feeding is sufficient to induce a molt when sucrose is provided subsequently. When larvae were fed casein-only diet for one or two days and then transferred to a non-nutritive diet, all larvae died within approximately seven to nine days, respectively, post feeding (data not shown). Thus, sucrose likely provides energy to keep the larvae alive until they initiate a molt.

### Ecdysone is Sufficient to Induce a Molt in Starved Animals

The inability of the larvae to molt in nutrient deprived conditions could either be due to reduced ecdysteroid sensitivity at the target tissues or to an alteration in ecdysteroid secretion from the prothoracic glands. To determine whether starved animals could molt in the presence of 20-hydroxyecdysone (20E), starved fourth instar larvae were injected with 25 µg of 20E every 12 hours. Most of the larvae initiated apolysis 36 hours after the first round of 20E injection even though they had only been fed a non-nutritive diet following the molt (Table 1). This suggests that the ability to respond to 20E is still intact in the starved animals. Thus, we conclude that the amino acids influence molting hormone secretion rather than the animal’s response to the molting hormone.

**Table 1:** The effect of 20-hydroxyecdysone injection on starved 4^th^ instar larvae.

		Number initiating apolysis					
Treatment	N	24 hrs	36 hrs	48 hrs	60 hrs	72+hrs	Died after 72+hrs
5 µl of 5 mg/ml 20E	6	1	4	1	0	0	0
Control (5 µl of water)	6	0	0	0	0	0	6

### Rapamycin Delays Molting

**Figure 3:**
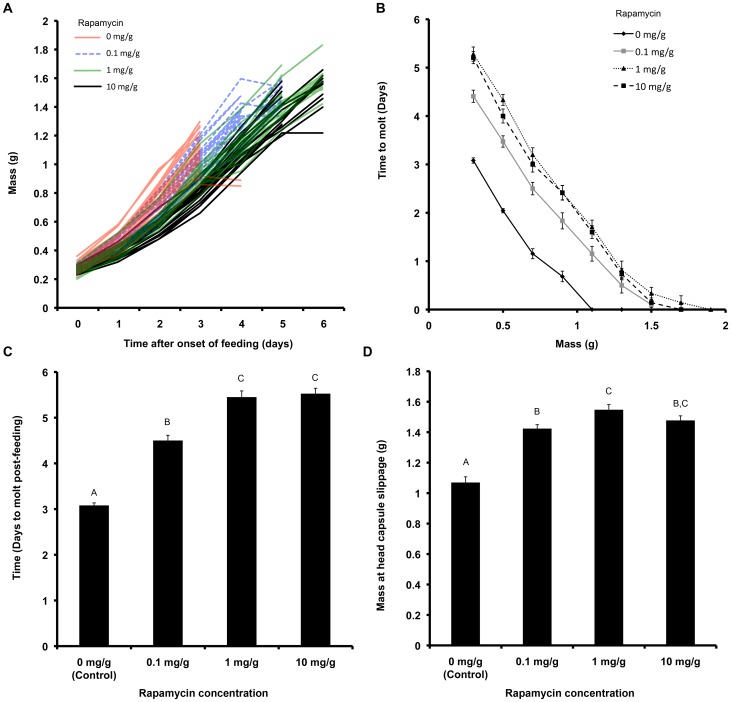
Effect of rapamycin on larval molt and time to molt. (A) Growth trajectories of larvae fed different concentrations of rapamycin. Differently colored lines represent different concentrations of rapamycin. (B) Time taken for larvae at various body sizes to initiate a molt. (C) Total time taken for larvae to initiate a molt. Different letters represent statistically significant differences (ANOVA with Tukey-Kramer HSD test; p<0.0001). All pairwise comparisons are significantly different from each other (p<0.0001) except between those treated with 1 mg/g and 10 mg/g rapamycin (p = 0.961). (D) Final body size at time of head capsule slip (n = 19–24 for each treatment). Different letters represent statistically significant differences (ANOVA with Tukey-Kramer HSD test; p<0.0001). All rapamycin fed larvae molted at a significantly larger size relative to the DMSO treated control larvae (p<0.0001). Larvae fed 1 mg/g rapamycin molted at a significantly larger size compared to those fed with 0.1 mg/g rapamycin (p = 0.0171). Error bars represent standard errors.

The above results indicated that amino acid intake was essential for initiation of synthesis or release of ecdysone in *M. sexta* larvae. Because previous studies have implicated the involvement of the amino acid sensitive TOR signaling in regulating the timing of metamorphosis in *Drosophila* [24], we investigated whether TOR signaling was involved in regulating the timing of the molt. We fed larvae normal diets supplemented with three different concentrations of rapamycin, a known inhibitor of TOR signaling. Control DMSO-treated larvae molted within approximately three days post initiation of feeding at a weight of approximately 1.1 g (Fig. 3A, C, D). Molting in rapamycin-treated larvae was delayed in a concentration–dependent manner (Fig. 3A–C). Many of these rapamycin-fed larvae molted at much larger weights compared to the controls (Fig. 3D) because they were able to ingest more nutrients during the 2–3 day delay in molting. At higher concentrations (1 mg/g and 10 mg/g rapamycin), rapamycin also decreased the growth rate (Fig. 3A). Taken together, these results suggest that amino acid mediated TOR signaling plays a role in determining the timing of the molt of a fourth instar into a fifth.

### Effect of Rapamycin and Nutrients on Growth of the Prothoracic Gland

**Figure 4:**
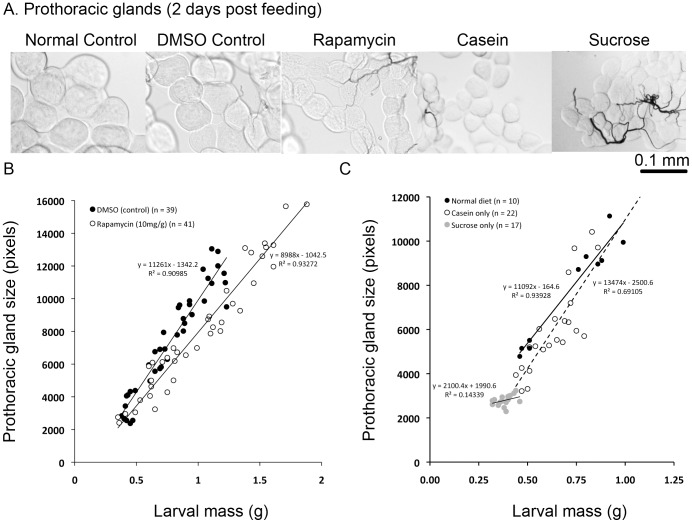
Effect of rapamycin and various nutrients on prothoracic gland growth. (A) Representative prothoracic glands, two days post feeding diets containing various nutrients. (B,C) Effect of rapamycin (B) and various nutrients (C) on prothoracic gland growth relative to body size. Prothoracic glands were dissected out from larvae at various weights. The size of prothoracic glands was determined by measuring the cross-sectional area of the glands. Larvae were transferred to the treated diets after being fed a non-nutritive diet for two days post head capsule slip at the end of the third instar. The glands were dissected after the larvae were fed the respective diets for at least one day.

The delay of molting caused by feeding rapamycin (Fig. 3A–C) indicated that the prothoracic gland might be rapamycin-sensitive and a potential sensor of amino acids required for molting. To investigate this, we isolated prothoracic glands from DMSO control and rapamycin-fed larvae at various weights and measured their sizes. When fed rapamycin, the prothoracic glands grew disproportionately slower relative to body size (Fig. 4A, B). Interestingly, however, the prothoracic glands of rapamycin–fed larvae ultimately reached the same size as those of DMSO control larvae as they continued to grow past the normal size at which a larva molts. This suggests an intriguing possibility that the prothoracic gland growth is correlated with a process that regulates the initiation of a molt, similar to what is observed in *Drosophila* [20], [21], [22].

We next sought to determine whether nutritional inputs also play a role in regulating prothoracic gland size. Sucrose fed larvae never molted (Fig. 1A), and this was accompanied by very little increase in size of the prothoracic glands (Fig. 4C). In contrast, casein fed larvae molted (Fig. 1A), and their prothoracic glands exhibited growth (Fig. 4C). The relative size of the prothoracic glands was smaller initially in the casein fed larvae; however, the relative size of the prothoracic glands was similar to that seen in larvae fed a normal diet later in the instar (Fig. 4C). Thus, nutritionally mediated mechanisms that regulate the growth of prothoracic glands might also regulate the timing of a molt.

### Growth of Body and Prothoracic Glands is Coupled Under Balanced Nutritional Inputs

**Figure 5:**
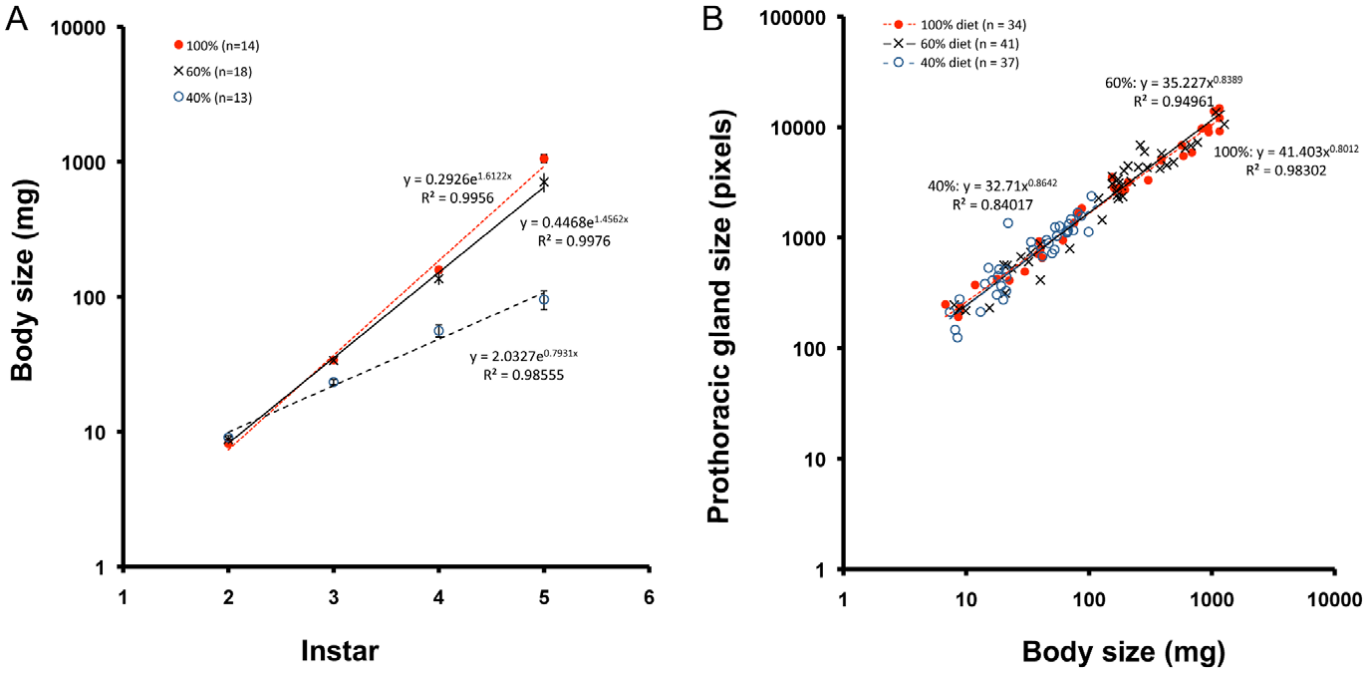
Effect of diet dilution on the timing of a molt and the growth of prothoracic glands. (A) Size of larvae at the time of head capsule slippage in larvae fed 40%, 60% or 100% diet from the first instar. (B) The relationship between body size and prothoracic gland size in larvae fed diluted diet from the first instar.

To determine how the growth of prothoracic glands might be correlated with body size, we fed larvae with 40%, 60% or 100% normal diet from the first instar and measured the growth trajectory of the prothoracic glands. Feeding larvae with diluted but nutritionally balanced diets caused larvae to molt at smaller sizes (Fig. 5A). Despite the smaller body size at the time of a molt, the prothoracic gland size and body size were found to retain the same size relationship even when larvae were fed a 40% diet (Fig. 5B). These results indicate that body growth and prothoracic gland growth are coupled by the same developmental process even when larvae are fed diluted diets. It is only when TOR signaling is disrupted that body growth and prothoracic gland growth become uncoupled.

### Rapamycin Suppresses 20E Release

**Figure 6:**
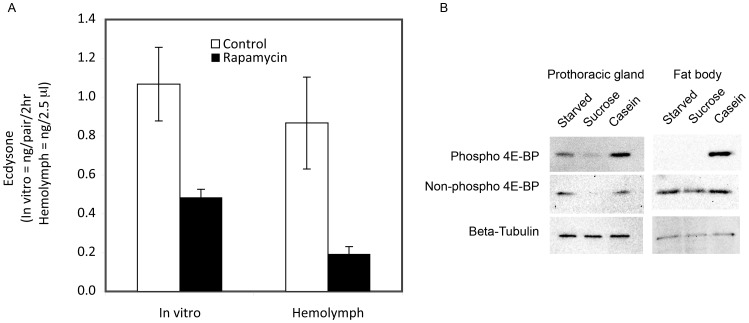
Effects of rapamycin and nutrients on ecdysone levels and 4E-BP phosphorylation states, respectively. (A) Effects of rapamycin treatment on ecdysone levels. Prothoracic glands and hemolymph from larvae fed 0.1 mg/ml rapamycin- or control DMSO-treated diets were isolated at average weights of 0.59±0.04 g and 0.61±0.02 g, respectively (Student’s t-test, *p* = 0.70, n = 8 larvae per group). Significant differences were observed between control and rapamyin-fed groups in hemolymph ecdysteroid levels, and in ecdysone secretion by isolated prothoracic glands (Student’s t-test, *p* = 0.014 and 0.009, respectively). Error bars indicate standard error for ecdysone secretion. (B) Effect of nutrients on 4E-BP phosphorylation states in the prothoracic glands and the fat body. In both the prothoracic glands and the fat body, the presence of amino acids led to elevated phosphorylated 4E-BP levels relative to sucrose fed larvae or starved larvae. In the prothoracic glands, phospho-4E-BP levels were also elevated in the starved larva relative to the sucrose fed larva.

The delayed timing of a molt and the reduced size of prothoracic glands in rapamycin-treated animals suggest that ecdysteroid release might be regulated in part by TOR signaling. To see if rapamycin could inhibit ecdysteroid release, we measured ecdysone titers in larvae fed diets containing 0.1 mg/g rapamycin. These larvae showed a delay in molting (Fig. 3A–C) and reduced prothoracic gland growth. As expected, day 3 rapamycin-treated larvae showed a statistically significant decrease in ecdysone production by the prothoracic glands and in hemolymph ecdysone levels, relative to control larvae of similar weights (Fig. 6A).

### Amino Acids are Required for Phosphorylation of 4E-BP

To see whether nutrients might influence TOR signaling, phosphorylation states of a downstream target of TOR signaling, 4E-BP, was analyzed in starved larvae and larvae fed casein-only or sucrose-only diets. In the prothoracic glands, the level of phosphorylated 4E-BP was elevated relative to those of starved and sucrose-fed larvae (Fig. 6B). Interestingly, prothoracic glands from starved larvae also showed slightly elevated phospho-4E-BP levels relative to those obtained from sucrose-fed larvae. Similar trends were observed for total 4E-BP levels. In the fat body, only casein fed larvae showed elevated phospho-4E-BP levels whereas the total 4E-BP levels were similar across the three treatments. Taken together, the presence of amino acids in the diet results in the phosphorylation of 4E-BP, indicating that amino acids activate TOR signaling in the fat body and the prothoracic glands.

## Discussion

In this study, we showed that the growth of the prothoracic glands and the initiation of a molt both require amino acid intake. Our finding that feeding rapamycin delays molting by suppressing ecdysone release suggests that TOR signaling may be a major contributor to the proper timing of a molt.

How nutritional signals regulate ecdysone production and release has recently begun to be elucidated. PTTH has been known to be a key signal required for ecdysone production and release. However, the responsiveness of the prothoracic glands to PTTH can vary with the physiological state. In *Manduca*, the prothoracic glands from starved fifth instar larvae are much less responsive to PTTH than those obtained from animals fed a nutrient rich diet, indicating that additional nutritionally mediated events must occur before the prothoracic glands can release ecdysone [23]. Recent studies indicate that, in addition to PTTH, insulin signaling also appears to play a key role in stimulating ecdysone release. In *Bombyx*, both insulin and PTTH influence ecdysteroidogenesis via PI3K/Akt signaling, whereas PTTH alone stimulates ERK signaling to influence ecdysteroidogenesis in the prothoracic glands [29], [30]. The lepidopteran homolog of insulin, bombyxin, is released from the brain and is particularly sensitive to nutritional inputs, especially glucose [31].

**Figure 7:**
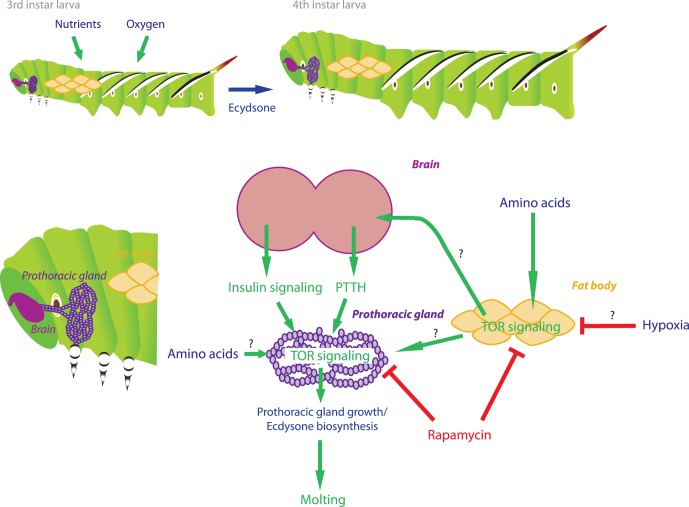
Model of how environmental cues might interact with TOR signaling to influence the timing of a molt.

Our study suggests that in addition to PTTH and insulin signaling, TOR signaling plays an important role in regulating ecdysone release/synthesis and the timing of a molt. Our study shows that feeding sucrose alone is insufficient to trigger a molt and allow prothoracic gland growth. Thus, amino acids must be present to either 1) stimulate or maintain production/release of additional factors from the brain, or 2) cause the prothoracic glands to become competent to respond to brain derived factors. Our rapamycin-treated animals showed depressed growth of the prothoracic glands relative to body size that in turn is correlated with a delay in the timing of a molt. We also showed that rapamycin represses ecdysone release in intact animals. The prothoracic glands scaled with body size when larvae were fed a diluted but balanced diet. Similar coupling between body size and prothoracic gland size has also been demonstrated in *Drosophila* [32]. Feeding rapamycin, however, disrupts this coupling, indicating that it most likely influences the brain or the prothoracic glands directly. These observations indicate that TOR signaling in the brain or prothoracic gland plays an essential role in ecdysone synthesis or release in *Manduca*. In *Drosophila,* TOR signaling in the prothoracic glands appears to regulate the timing of metamorphosis by influencing the production/release of ecdysone [24]. *In vitro* incubation of prothoracic glands with rapamycin also results in suppressed PTTH-stimulated ecdysteroidogenesis in the silkworm *Bombyx mori* [33]. Thus, the function of TOR signaling in the production and release of ecdysone may be conserved among different insects at different developmental stages.

We also found that the growth rate became progressively depressed at higher concentrations of rapamycin. Thus, rapamycin can affect both the growth of the prothoracic glands directly and indirectly by altering the growth of the whole body at higher concentrations. We therefore think that in addition to increasing the competency of prothoracic glands to produce and release ecdysone, TOR signaling might also act on some remote tissue upstream of prothoracic glands to influence the timing of a molt. The fat body has been shown to act as a nutrient sensor in *Drosophila* [34], and the presence of a fat body derived ecdysteroid stimulatory factor has been proposed by several studies done in Lepidoptera [35], [36]. Thus, the fat body may integrate nutritional status of the larva and send signals to the brain and/or the prothoracic glands to regulate the timing of a molt (Fig. 7). Insulin signaling, known to be mediated in part via TOR signaling [37], is a possible candidate.

A recent study on the physiological underpinnings of critical weight has demonstrated that oxygen levels in the body might be a key determinant of the critical weight [17]. Under hypoxic conditions, *Manduca* larvae attain critical weight at a smaller size relative to those reared under normoxic conditions. Because the trachea do not grow during the intermolt period, it has been proposed that as the larvae grow, tissue oxygenation is diminished, signaling a yet-undetermined oxygen sensor to initiate a molt. How the information about the internal oxygen levels is relayed to the brain and the prothoracic gland is not known.

Recent studies have begun to show interactions between hypoxia and nutrition in regulating physiological processes [38]. Hypoxia-induced signaling pathway can act in parallel with the nutritionally sensitive insulin signaling to influence a physiological or developmental mechanism [38]. In particular, hypoxia has been shown to inhibit TOR signaling [39], [40], and in flies, hypoxia-induced suppression of TOR signaling has been shown to be linked to decreased body size [41]. Thus, TOR kinase activity might be a common mechanism for inhibition of growth, whether by hypoxia or by rapamycin. However, while hypoxia causes premature molting by lowering the critical weight, rapamycin feeding causes a delay in the molting time. Possibly, this seemingly contradictory observation might be explained by the fact that hypoxia might only influence tissues located far from the trachea, where oxygen limitation becomes an issue; prothoracic glands are located close to the spiracle and may not experience severe oxygen limitation in response to hypoxia. In contrast, ingestion of rapamycin may affect all tissues in the body. Alternatively, it is also possible that hypoxia influences TOR-independent pathways, either directly or through an effect on upstream regulatory factors. Future studies on the molecular mechanisms by which hypoxia influences the timing of a molt should shed light on this matter.

Puberty in humans is marked by endocrine changes in response to increased pulsatile release of gonadotropin releasing hormone, GnRH, from the hypothalamus, in turn triggered by the hypothalamic regulatory peptide, kisspeptin. The timing of these endocrine changes and initiation of puberty are thought to be regulated by some type of integrator by which environmental conditions are assessed and combined with an unknown developmental timer [3], [42]. While little is known about the developmental timer, a variety of environmental factors are known to modulate when the release of kisspeptin and GnRH begins to accelerate [3], [43]. Nutritional conditions play important roles in regulating the timing of entry into puberty: malnutrition delays the onset of puberty and obesity leads to precocious puberty [44], [45], [46]. Thus, nutrients play an important role in regulating developmental transitions in both insects and humans. A further intriguing similarity between molting regulation and puberty is seen by the involvement of TOR signaling in both instances. mTOR appears to act upstream of kisspeptin and GnRH in vertebrates [47], [48], [49], [50]. Our current study, along with the previous study on *Drosophila* metamorphosis by Layalle et al (2008), shows that TOR signaling plays a key role in modulating the timing of ecdysone secretion. Thus, TOR signaling may play important roles during major developmental events in both insects and vertebrates.

## Supporting Information

Table S1 **Ingredients for the various diets used in this study.**
(PDF)
