# Supplementary material for: Amino Acids and TOR Signaling Promote Prothoracic Gland Growth and the Initiation of Larval Molts in the Tobacco Hornworm Manduca sexta
Source: PLoS One. 2012 Sep 12;7(9):e44429. doi: 10.1371/journal.pone.0044429 (PMC3440373; doi:10.1371/journal.pone.0044429)
Supplement: Table S1 — Ingredients for the various diets used in this study. (PDF) [file pone.0044429.s001.pdf]

Supporting information

Table S1: Ingredients for the various diets used in this study.

Normal diet = Ingredients A + B

Negative control diet = Ingredient B

Casein only diet = Casein + Ingredient B

Sucrose only diet = Sucrose + Ingredient B

Sucrose + casein diet = (8%- % casein) Sucrose + % Casein + Ingredient B

Ingredient A. Nutritive base

INGREDIENTS	AMOUNT
Wheat germ	107.6 g
Casein	48.4 g
Sucrose	43.0 g
Torula yeast	21.5 g
Cholesterol	4.71 g

Ingredient B. Non-nutritive base

INGREDIENTS	AMOUNT
Distilled water	1000 ml
Gelcarin	15.7 g
Wessons salt	16.1 g
Sorbic acid	2.7 g
Methyl paraben	1.3 g
Ascorbic acid	6.7 g
Streptomycin	0.27 g
Kanamycin	0.07 g
36.5% formalin	8.6 ml
**Vitamin mix**<br><br>Nicotinic acid<br>Riboflavin<br>Thiamine<br>Pyriodoxine<br>Folic acid<br>Biotin	**Dissolved in 10ml water**<br><br>13.5 mg<br>6.73 mg<br>3.14 mg<br>3.14 mg<br>3.14 mg<br>0.27 mg
